# Dual-scope method for hemostasis during transbronchial lung cryobiopsy: A two-patient case report

**DOI:** 10.1097/MD.0000000000051044

**Published:** 2026-10-09

**Authors:** Ryutaro Hanawa, Satoshi Okamori, Mikito Suzuki, Keisuke Asakura, Mitsutomo Kohno, Ichiro Nakachi

**Affiliations:** a Department of General Thoracic Surgery, Saiseikai Utsunomiya Hospital, Tochigi, Japan; b Pulmonary Division, Department of Internal Medicine, Saiseikai Utsunomiya Hospital, Tochigi, Japan; c Department of Thoracic Surgery, Tokyo Metropolitan Cancer and Infectious Diseases Center Komagome Hospital, Tokyo, Japan; d Division of Thoracic Surgery, Department of Surgery, Keio University School of Medicine, Tokyo, Japan; e Department of General Thoracic Surgery, Saitama Medical Center, Saitama Medical University, Saitama, Japan; f Department of Respiratory Medicine, Saiseikai Utsunomiya Hospital, Utsunomiya, Tochigi, Japan.

**Keywords:** balloon occlusion, bronchoscopy, case report, cryobiopsy, hemostasis

## Abstract

**Rationale:**

Bleeding during transbronchial lung cryobiopsy can be clinically consequential. Conventional balloon occlusion and sequential 2-scope techniques may limit visualization or require bronchoscope exchange. We developed a dual-scope method in which an ultrathin bronchoscope for sampling and a therapeutic bronchoscope for balloon control are positioned simultaneously.

**Patient concerns:**

An 84-year-old man with a 0.6-cm peripheral right upper lobe nodule after prior resection of squamous cell carcinoma and a 55-year-old woman with a 1.2-cm peripheral right lower lobe nodule during surveillance for peritoneal cancer required tissue diagnosis.

**Diagnoses:**

In Case 1, a single cryobiopsy specimen was diagnostically adequate, showed no crush artifact, and revealed nonkeratinizing squamous cell carcinoma. Compared with the previously resected keratinizing squamous cell carcinoma, the lesion was considered more likely to represent a new primary lung cancer, although this could not be determined definitively from the bronchoscopic specimen alone. In Case 2, a single diagnostically adequate specimen without crush artifact showed acinar-forming adenocarcinoma; subsequent surgical resection confirmed primary lung adenocarcinoma, pT1aN0M0, stage IA1.

**Interventions:**

A 1.1-mm cryoprobe was advanced through the ultrathin bronchoscope, while a balloon catheter was positioned through the working channel of the therapeutic bronchoscope. Immediately after specimen retrieval, the balloon was inflated prophylactically under direct endoscopic observation.

**Outcomes:**

Balloon occlusion was maintained for 5 minutes in Case 1 and 3 minutes in Case 2. After balloon deflation, only a few milliliters of bleeding were observed in each case and resolved within a few seconds with suction alone. No repeat balloon inflation, topical hemostatic agent, or interruption of the procedure was required; bleeding was classified as Nashville Bleeding Scale grade 1 in both cases. Oxygen saturation remained 99% and 98% to 99%, respectively. Neither patient developed pneumothorax, delayed bleeding, or readmission due to a bronchoscopy-related complication.

**Lessons:**

These cases demonstrate the technical feasibility of simultaneous dual-scope positioning and real-time balloon management during Transbronchial lung cryobiopsy. They do not establish safety, reliability, or superiority over existing techniques; larger prospective studies using standardized bleeding assessment are required.

## 1. Introduction

Bleeding is a recognized and potentially serious complication of transbronchial lung cryobiopsy (TBLC), and rare fatal events have been reported.^[1,2]^ Prophylactic balloon occlusion and the 2-scope technique are commonly used to limit central airway flooding after cryobiopsy.^[3–5]^ However, selective balloon placement can be technically difficult, and sequential bronchoscope exchange creates a period during which the airway is not directly visualized.^[6]^ The optimal hemostatic workflow for TBLC of peripheral pulmonary lesions therefore remains uncertain.^[7]^

We developed a dual-scope method in which an ultrathin bronchoscope used for sampling and a therapeutic bronchoscope used for balloon control are positioned simultaneously through the mouth. This report describes the technical application of the method in 2 clinically distinct patients. The report was prepared in accordance with the CARE guidelines.^[8]^

## 2. Methods

### 2.1. Dual-scope procedure

An ultrathin bronchoscope (BF-MP290F; Olympus, Tokyo, Japan) was used for sampling, and a therapeutic bronchoscope (BF-1TH1200; Olympus) was used for balloon control and hemostatic observation. A single-use 1.1-mm cryoprobe (CRYO2; ERBE Elektromedizin GmbH, Tuebingen, Germany) was advanced through the ultrathin bronchoscope, and a balloon catheter (B5-2C; Olympus) was advanced through the working channel of the therapeutic bronchoscope. Midazolam and fentanyl were administered for sedation and analgesia, with propofol added when necessary; 4% lidocaine was used for topical anesthesia. The procedure comprised the following steps: the operator, hemostasis assistant, and balloon assistant were positioned at the patient’s head (Fig. 1A); the airway was examined with the therapeutic bronchoscope, which was then exchanged for the ultrathin bronchoscope and advanced to the target lesion; the therapeutic bronchoscope was reintroduced orally alongside the ultrathin bronchoscope (Fig. 1B); the balloon catheter was advanced through the therapeutic bronchoscope and positioned in the target bronchus (Fig. 1C); cryobiopsy was performed, after which the balloon was inflated prophylactically and the ultrathin bronchoscope was withdrawn under direct endoscopic observation (Fig. 1D); and the balloon was deflated after a predefined interval and the airway was assessed for ongoing bleeding.

**Figure 1.**
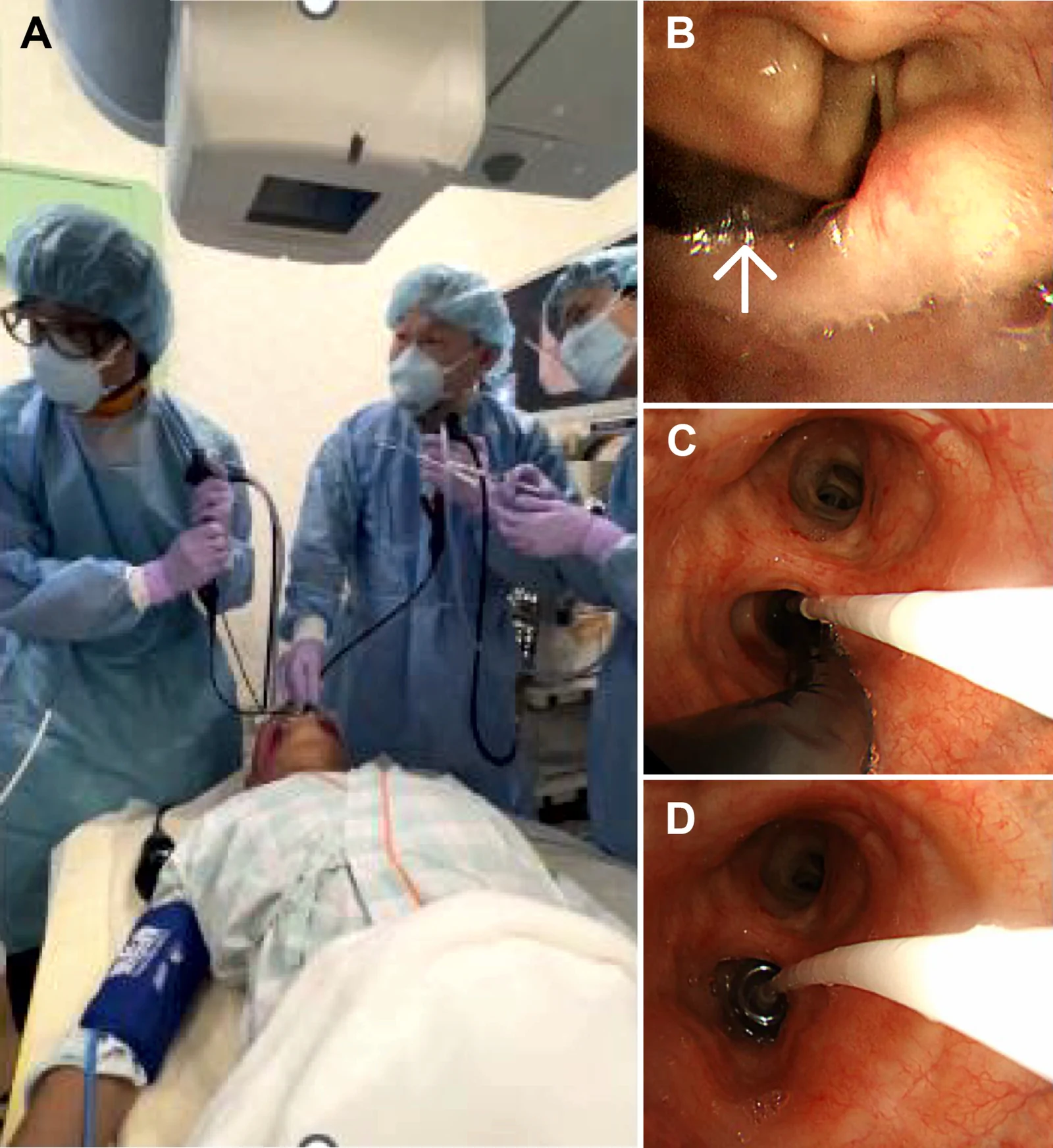
(A) Staffing setup for the dual-scope method. From left to right: the operator, hemostasis assistant, and balloon assistant. (B) Dual-scope positioning at the level of the vocal cords. The ultrathin bronchoscope (single arrow) passes through the vocal cords first. (C) The balloon catheter is guided through the working channel of the therapeutic bronchoscope to the bronchus selected for occlusion. (D) After removal of the ultrathin bronchoscope, the balloon is inflated under direct endoscopic visualization, allowing immediate confirmation of balloon position without bronchoscope exchange.

### 2.2. Ethical considerations

Formal ethical review was not undertaken because both procedures were performed as part of routine clinical care within the scope of Japan’s national health insurance system, involved no off-label use, and this manuscript reports 2 anonymized cases. Written informed consent was obtained from both patients for publication of the clinical information and images in this report.

## 3. Case presentations

### 3.1. Case 1

An 84-year-old man (height, 157 cm; weight, 54 kg) with heart failure and chronic obstructive pulmonary disease had undergone video-assisted thoracoscopic right lower lobectomy for keratinizing squamous cell carcinoma in April 2020. Surveillance computed tomography in July 2024 revealed a 0.6-cm solid peripheral nodule in right S3. Selective fixation of a balloon in the right upper lobe using the conventional approach was considered difficult; therefore, the dual-scope method was selected. TBLC was performed in August 2024. Balloon occlusion of the right upper lobe bronchus was maintained for 5 minutes, and the procedure time was 31 minutes with oxygen saturation maintained at 99% (Fig. 2). After balloon deflation, only a few milliliters of bleeding were observed and resolved within a few seconds with suction alone. Repeat balloon inflation, topical hemostatic agents, and interruption of the procedure were not required; this was classified as Nashville Bleeding Scale (NBS) grade 1.^[9]^ A single cryobiopsy specimen was sufficient for histopathological evaluation and showed no crush artifact. The specimen revealed nonkeratinizing squamous cell carcinoma. Because the previously resected tumor showed keratinization, the new lesion was considered more likely to represent a new primary lung squamous cell carcinoma rather than recurrence, although this distinction could not be established definitively from the bronchoscopic specimen alone. No pneumothorax, delayed bleeding, or readmission due to a bronchoscopy-related complication occurred. The patient subsequently underwent stereotactic radiotherapy (48 Gy in 4 fractions) and has been followed up for 1 year and 11 months after treatment.

**Figure 2.**
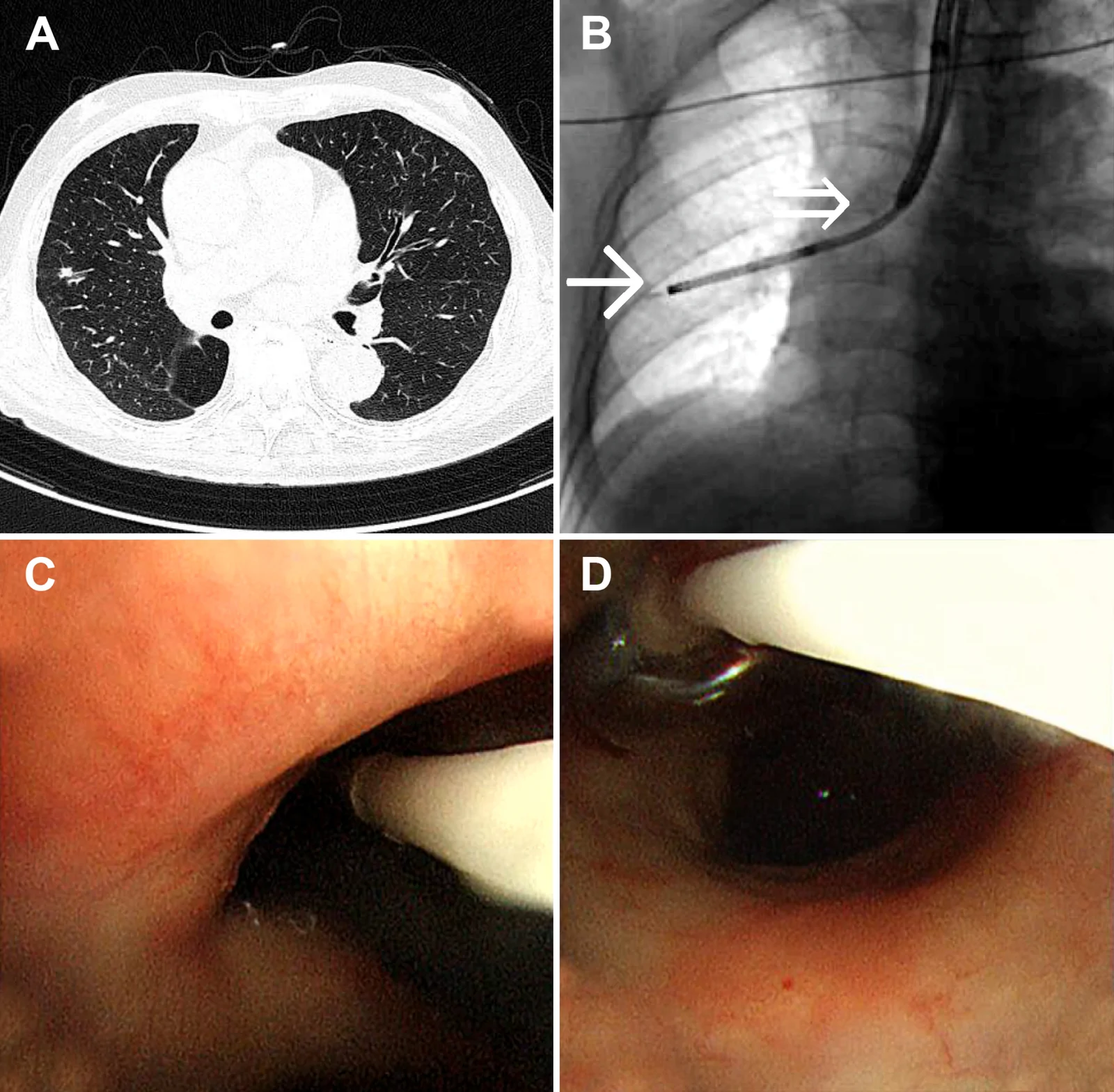
Case 1. (A) A 0.6-cm solid nodule in the right upper lobe B3aiβ. (B) Fluoroscopic image during cryobiopsy. The single arrow indicates the tip of the ultrathin bronchoscope, and the double arrow indicates the tip of the therapeutic bronchoscope. (C) Bronchoscopic view immediately before cryobiopsy showing the ultrathin bronchoscope and balloon catheter simultaneously. (D) After removal of the ultrathin bronchoscope, the balloon is inflated under direct endoscopic visualization, permitting confirmation of its position and timing of inflation.

### 3.2. Case 2

A 55-year-old woman (height, 152 cm; weight, 88 kg) with bronchial asthma and a history of treatment for peritoneal serous carcinoma underwent surveillance computed tomography in May 2024, which revealed a 1.2-cm solid peripheral nodule in right S8. Owing to her short stature, obesity, and relatively narrow tracheobronchial tree, tracheal intubation was considered potentially difficult; therefore, the dual-scope method without routine tracheal intubation was selected. TBLC was performed in August 2024. Balloon occlusion of the right basal bronchus was maintained for 3 minutes, and the procedure time was 31 minutes with oxygen saturation maintained at 98% to 99% (Fig. 3).After balloon deflation, only a few milliliters of bleeding were observed and resolved within a few seconds with suction alone. Repeat balloon inflation, topical hemostatic agents, and interruption of the procedure were not required; this was classified as NBS grade 1. A single cryobiopsy specimen was sufficient for histopathological evaluation and showed no crush artifact. The specimen showed acinar-forming adenocarcinoma. Because the previously treated peritoneal carcinoma was serous carcinoma, the pulmonary lesion was considered more likely to represent a separate primary lung adenocarcinoma rather than metastasis. The patient underwent robot-assisted right lower lobectomy with ND2a-1 lymph node dissection in September 2024, and the resection specimen confirmed lung adenocarcinoma, pT1aN0M0, stage IA1. No pneumothorax, delayed bleeding, or readmission due to a bronchoscopy-related complication occurred. At 1 year and 10 months after surgery, no recurrence of lung cancer had been observed. She was receiving chemotherapy for recurrent peritoneal dissemination of peritoneal serous carcinoma.

**Figure 3.**
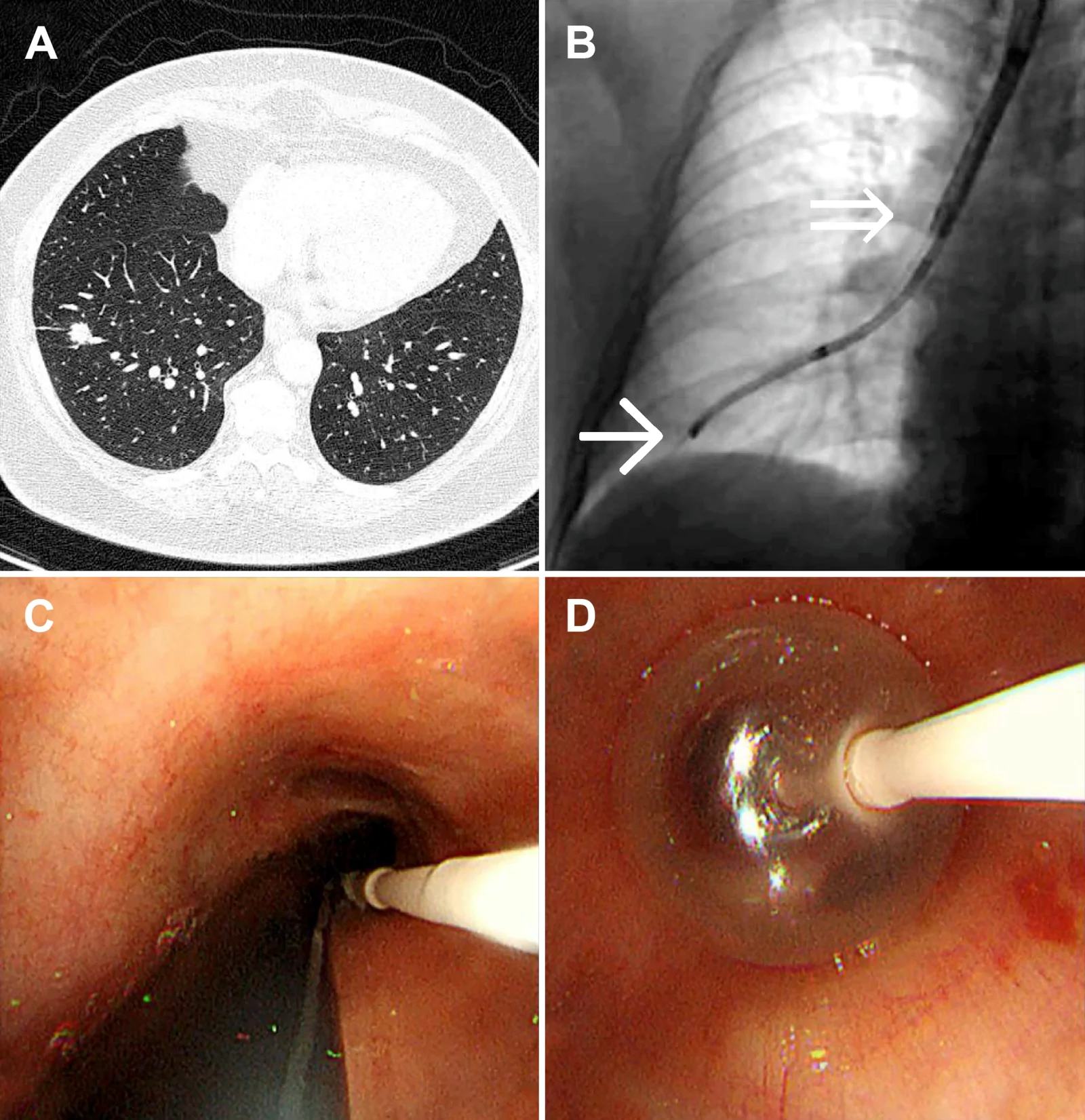
Case 2. (A) A 1.2-cm solid nodule in peripheral right lower lobe B8a. (B) Fluoroscopic image during cryobiopsy. The single arrow indicates the tip of the ultrathin bronchoscope, and the double arrow indicates the tip of the therapeutic bronchoscope. (C) Bronchoscopic view immediately before cryobiopsy showing the ultrathin bronchoscope and balloon catheter simultaneously. (D) After removal of the ultrathin bronchoscope, the balloon is inflated under direct endoscopic visualization.

The clinical timelines and procedural outcomes of both cases are summarized in Table 1.

**Table 1:** Clinical timelines and key procedural outcomes of the 2 cases.

Date/clinical phase	Case 1	Case 2
April 2020	Video-assisted thoracoscopic right lower lobectomy for keratinizing squamous cell carcinoma	–
May 2024	–	Surveillance CT for peritoneal carcinoma revealed a 1.2-cm peripheral nodule in right S8
July 2024	Surveillance CT revealed a 0.6-cm peripheral nodule in right S3	–
August 2024: TBLC	Dual-scope TBLC; balloon occlusion of the right upper lobe bronchus for 5 min; procedure time, 31 min; SpO_2_ maintained at 99%; one diagnostically adequate specimen without crush artifact	Dual-scope TBLC; balloon occlusion of the right basal bronchus for 3 min; procedure time, 31 min; SpO_2_ maintained at 98% –99%; one diagnostically adequate specimen without crush artifact
Immediate and postprocedural outcomes	After balloon deflation, a few milliliters of bleeding resolved within seconds with suction alone; no repeat balloon inflation, topical hemostatic agent, or procedure interruption; NBS grade 1; no pneumothorax, delayed bleeding, or bronchoscopy-related readmission	After balloon deflation, a few milliliters of bleeding resolved within seconds with suction alone; no repeat balloon inflation, topical hemostatic agent, or procedure interruption; NBS grade 1; no pneumothorax, delayed bleeding, or bronchoscopy-related readmission
Pathological diagnosis	Nonkeratinizing squamous cell carcinoma	Acinar-forming adenocarcinoma
August 2024	Stereotactic radiotherapy (48 Gy in 4 fractions)	–
September 2024	–	Robot-assisted right lower lobectomy with ND2a-1 lymph node dissection; final diagnosis: lung adenocarcinoma, pT1aN0M0, stage IA1
July 2026	Follow-up 1 year 11 months after radiotherapy; no bronchoscopy-related complications	Follow-up 1 year 10 months after surgery; no lung cancer recurrence; chemotherapy for recurrent peritoneal dissemination of peritoneal serous carcinoma

CT=computed tomography, NBS=Nashville Bleeding Scale, SpO_2_=oxygen saturation, TBLC=transbronchial lung cryobiopsy.

## 4. Discussion

These 2 cases demonstrate the technical feasibility of placing an ultrathin sampling bronchoscope and a therapeutic bronchoscope simultaneously during TBLC. The method permitted direct visualization of balloon position and inflation while the sampling bronchoscope was withdrawn. In Case 1, channel-guided balloon placement enabled selective occlusion of the right upper lobe bronchus, a location where external balloon guidance may be difficult. In Case 2, the procedure was completed without tracheal intubation despite anticipated difficulty in establishing an artificial airway. These observations describe potential procedural features rather than comparative advantages.

The method may offer 3 practical features in selected patients: continuous endoscopic observation during specimen retrieval and balloon inflation; channel-guided, selective balloon positioning; and avoidance of routine tracheal intubation. However, the present report does not establish that these features improve safety, oxygenation, hemostatic reliability, or procedural efficiency compared with conventional balloon occlusion or sequential 2-scope techniques. The stable oxygenation observed in these 2 patients may reflect patient selection, lesion location, sedation, and selective lobar occlusion, and should not be interpreted as evidence of superiority.

Bleeding severity was retrospectively assessed using the NBS, which grades interventions performed in response to active bleeding.^[9]^ Prophylactic balloon placement is not itself graded; balloon reinflation in response to continued bleeding constitutes a higher-grade intervention. In both cases, after planned balloon deflation, only a few milliliters of bleeding were observed and resolved within a few seconds with suction alone, without balloon reinflation or other hemostatic treatment. Both events were therefore classified as NBS grade 1. These observations document the immediate procedural course but do not establish a lower bleeding risk than that associated with other techniques.

A single cryobiopsy specimen was obtained in each case, and both specimens were diagnostically adequate without crush artifact. In Case 1, the specimen showed nonkeratinizing squamous cell carcinoma, whereas the prior resection specimen had shown keratinizing squamous cell carcinoma; this suggested a new primary lesion, although the distinction from recurrence could not be made definitively from the bronchoscopic specimen alone. In Case 2, acinar-forming adenocarcinoma differed morphologically from the previous peritoneal serous carcinoma, and subsequent lobectomy confirmed primary lung adenocarcinoma, pT1aN0M0, stage IA1. These 2 diagnostic results should not be interpreted as an estimate of diagnostic yield.

This report has several limitations. It includes only 2 selected patients from a single institution, has no comparator group, and was not designed to assess safety or efficacy. Bleeding severity and oxygenation outcomes were not prospectively defined, and the NBS grading was applied retrospectively. Although neither patient developed pneumothorax, delayed bleeding, or bronchoscopy-related readmission during follow-up, the sample is too small to characterize complication rates. The present report should therefore be interpreted as a proof-of-concept demonstrating the technical feasibility of the dual-scope method rather than evidence of superiority over existing techniques. Prospective comparative studies are warranted to determine whether this approach improves procedural safety, bleeding control, or diagnostic performance.

Patient perspective: The patients’ perspectives on the procedure were not formally collected.

## 5. Conclusions

In 2 selected patients, the dual-scope method was technically feasible and allowed simultaneous endoscopic observation and targeted balloon positioning during TBLC. These preliminary observations do not establish safety, reliability, or superiority over existing methods. Larger prospective comparative studies are required.

## Acknowledgments

The authors thank the medical staff and collaborators at Saiseikai Utsunomiya Hospital, Tokyo Metropolitan Cancer and Infectious Diseases Center Komagome Hospital, and the participating institutions for their support.

## Author contributions

**Conceptualization:** Ryutaro Hanawa, Satoshi Okamori.

**Supervision:** Satoshi Okamori, Mikito Suzuki, Keisuke Asakura, Mitsutomo Kohno, Ichiro Nakachi.

**Writing – original draft:** Ryutaro Hanawa.

**Writing – review & editing:** Satoshi Okamori, Mikito Suzuki, Keisuke Asakura, Mitsutomo Kohno, Ichiro Nakachi.
